# The Antiplasmodial Potential of Medicinal Plants Used in the Cameroonian Pharmacopoeia: An Updated Systematic Review and Meta-Analysis

**DOI:** 10.1155/2022/4661753

**Published:** 2022-10-08

**Authors:** Arnaud Gabin N. Tepa, Panthaleon Ambassa, Lawrence S. Ayong, Prosper Cabral Biapa Nya, Constant Anatole Pieme

**Affiliations:** ^1^Department of Biochemistry, Faculty of Medicine and Biomedical Sciences, P.O. Box 1364, University of Yaoundé 1, Yaounde, Cameroon; ^2^Department of Organic Chemistry, Faculty of Sciences of the University of Yaoundé 1, P.O. Box 812, Yaounde, Cameroon; ^3^Public Health and Epidemiology Unit, Centre Pasteur Du Cameroun, P. O. Box 1274, Yaoundé, Cameroon; ^4^Department of Biochemistry, Faculty of Sciences, University of Dschang, Dschang, Cameroon

## Abstract

Malaria is a real public health problem. It is the leading cause of morbidity and mortality in the world. Research in herbal medicine has so far shown that the use of plants against malaria is not to be neglected. This review aims to highlight the antiplasmodial potential of Cameroonian plants. In order to achieve this objective, we conducted a bibliographic search in April 2022 using the PubMed search engine. This research included both the published and unpublished studies. A narrative approach was used to describe the antiplasmodial potential of the various species of plants investigated. Quantitative data were analyzed using *R* studio 4.1.1 software and random effects model was used to estimate the effect size. The research of the antiplasmodial activity of Cameroonian plants dates back to 2000. This area of research has since provided extensive data to indicate the antiplasmodial potential of several plants, most of which originate from the central region. Despite the heterogeneity observed between the different plant families studied in Cameroon for their *in vitro* antiplasmodial effect, there is strong evidence that 17 active compounds from these plants would be ideal candidates for the synthesis of new antimalarial drugs. The *Dacryodes edulis* species could be considered as the best natural alternative in the treatment of uncomplicated malaria according to its properties. It is clear that the traditional Cameroonian pharmacopoeia has many species that contain compounds with antiplasmodial activity. More studies need to be conducted to explore the multitude of unexplored plants that are used in traditional medicine. These studies should take into account the nature of the cell model used for cytotoxicity assessment.

## 1. Introduction

Malaria remains a global public health problem with about 228 million cases worldwide and 213 million cases (93%) recorded in Africa [1]. Multiple control strategies against this endemic, including vector control through the widespread use of long-lastinginsecticide-treated nets or indoor residual spraying on the one hand and chemoprevention on the other hand, have so far largely contributed to reduce the incidence of malaria in the world [2, 3]. Unfortunately, these advances are constantly threatened by the emergence of resistance not only of the vectors to the insecticides used [4–6], but also of the parasite to the drugs. In the 1990s, the emergence of chloroquine resistance was associated with a dramatic increase in malaria mortality [7]. At the end of the last century, introduction of the artemisinin combination therapies (ACTs) provided a much needed, highly efficacious antimalarial treatment, which became the first-line treatment for uncomplicated falciparum malaria in all endemic countries [8]. The extremely rapid development of resistance to many antimalarials, and even the most recent, such as ACTs in five countries of the Greater Mekong subregion [9, 10] and in Africa [11, 12], justifies continued research on the factors causing this resistance. Like antibiotic resistance, antimalarial drug resistance is caused by the massive and uncontrolled use of certain molecules that could lead to a selection of resistant strains of Plasmodium over time. Diversification of effective antimalarial drugs would therefore be a solution to significantly reduce the rapid progression of resistance and thus the malaria-related mortality.

It has been highlighted that the richness of plant biodiversity and the knowledge of traditional therapies are likely to open new avenues for antimalarial therapy [13]. This was for example the case of quinine and artemisinin, which are the two currently prescribed antimalarials from medicinal plants, traditionally used in their country of origin against fevers and malaria. Quinine is from the bark of a tree from the flanks of the *Andean cordillera* (*Cinchona calisaya* and other species of *Cinchona*) [14] and artemisinin is from a herb native to China, *Artemisia annua* [15]. The search for new antimalarial drugs could therefore be undertaken within plant biodiversity using ethnopharmacology. Through this approach, the potential antimalarial activity of plants could guide the scientific community towards more in-depth research. This review aims to highlight the antiplasmodial potential of the plants of the Cameroonian pharmacopeia while evaluating their ability to inhibit *in vitro* chloroquine-sensitive and chloroquine-resistant strains with the least cytotoxicity possible.

## 2. Methods

The proposed systematic review was conducted in accordance to the Cochrane Handbook [16] and PRISMA statement (i.e., Preferred Reporting Items for Systematic Reviews and Meta-analyses [17]).

The following research question was formulated to address the literature and outline the search strategy: are Cameroonian plants species or family able to be more effective with low toxicity *in vitro* against Plasmodium resistant-chloroquine strains compare to sensitive-chloroquine one?

### 2.1. Search Strategy

An electronic search on the PubMed database was performed up to April 06^th^, 2022. The search strategy aimed to locate both published and unpublished studies. The computer database search in PubMed was performed using the following keywords: (((((((“antimalarial activity”[Body—All Words] OR “antiplasmodial activity”[Body—All Words]) OR antimalarial[Abstract]) OR antiplasmodial[Abstract]) OR antimalarial[Title]) OR antiplasmodial[Title]) AND (“*in vitro*”[Body—All Words] OR *in vitro* [Body—All Words])) AND Cameroon[Body—All Words]) AND (IC_50_[Abstract] OR IC_50_[Body—All Words]). No publication year or language limit was considered.

### 2.2. Selection, Inclusion, and Exclusion Criteria

Following the search, all identified citations were collected and uploaded into the Zotero software and duplicates were removed. Titles and abstracts were then screened by one reviewer for assessment against the inclusion criteria for the review. Potentially relevant studies were retrieved in full and their citation details were imported into the Rayyan software [18]. The full text of selected citations was assessed in detail against the inclusion criteria by one reviewer. Reasons for exclusion of full text studies that do not meet the inclusion criteria were recorded and reported in the systematic review. Review considered studies that included Cameroonian plants assessed for their *in vitro* antiplasmodial activity. Only primary studies assessing the *in vitro* 50% inhibitor concentration (IC_50_) were included in the review. All review articles were excluded. The results of the search were reported in full in the final systematic review and presented in a Preferred Reporting Items for Systematic Reviews and Meta-analyses (PRISMA) flow diagram [17].

### 2.3. Data Extraction

Data was extracted from papers and included in the review by one reviewer using a data extraction tool developed by the reviewer. The data extracted included specific details about the plants species, family, place of harvest, part use, extract, metabolite, used parasite, cell model, IC_50_, 50% cytotoxic concentration (CC_50_), standard deviation of each quantitative variable, sample size. The selectivity index (SI) of each extract was calculates as follows: CC_50_/IC_50_. The extracted data was presented in tabular form to align with the objective of this review. A narrative summary accompanied the tabulated results and described how the results relate to the reviews objective and question.

### 2.4. Risk of Bias Assessment

Eligible studies were critically appraised by one reviewer considering a score described in previous systematic reviews of *in vitro* studies [19]. The description of the following parameters was checked in each study: clear extraction method, appropriate *in vitro* method for antimalarial activity, appropriate number of replicate, resistant vs. sensitive Plasmodium strain comparison, appropriate *in vitro* method for cytotoxicity, culture of Plasmodium and control cells in the same condition, availability of all required outcome (IC_50_, CC_50_ and SDs) and quality control valid. If the parameter was described on the text, the study received a “yes” on that specific parameter, otherwise it had a “no.” The risk of bias was classified according to the sum of “yes” received as follows: 1–3 = high, 4–5 = medium, 6–8 = low risk of bias. The results of critical appraisal is reported in narrative form and in a table. Only the low risk of bias studies was included in the meta-analysis.

### 2.5. Data Synthesis

Selected studies were pooled in statistical meta-analysis using *R* Studio software V4.1.1. Effect sizes were expressed as standard mean difference (SMD) for selectivity index and 95% confidence level was considered for analysis. Heterogeneity was assessed statistically using the standard Chi-squared and I squared tests. Statistical analyses were performed using random effects model [20]. Plants or metabolites with SMD <0.8 were considered as good antiplasmodial drug candidate against both chloroquine/multi-drug-resistant (experimental) and sensitive (control) *Plasmodium* strain. Forest plots were created to illustrate the meta-analysis. Where statistical pooling was not possible, the findings were presented in narrative form including tables to aid in data presentation where appropriate. A funnel plot was generated to assess publication bias. Egger's test for funnel plot asymmetry was performed where appropriate.

## 3. Results

### 3.1. Search and Selection

**Figure 1:**
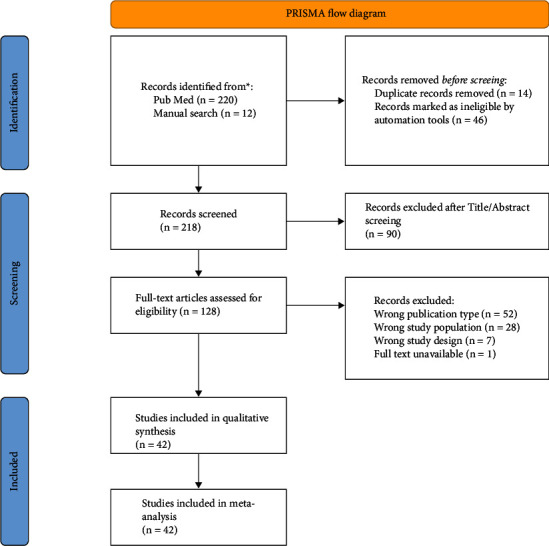
Flowchart diagram of study selection according to PRISMA statement.

A total of 220 articles were retrieved by automatic search on PubMed. Manual search based on reference screening completed our search with another 12 articles (Figure 1). From the 232 articles downloaded, 14 were duplicates and were removed, 46 were reviewed and therefore automatically excluded. The titles and abstracts of 218 articles were screened, 90 were excluded as not being on the topic of interest. Of the 128 eligible articles, 86 were excluded for various reasons such as: study population (28), type of publication (52), study design (7), and unavailability of full text (1). The remaining 42 articles were included in the qualitative analysis and assessed for risk of bias. Only 10 articles with low risk of bias were included in meta-analysis (Table S1).

### 3.2. Descriptive Analysis

**Figure 2:**
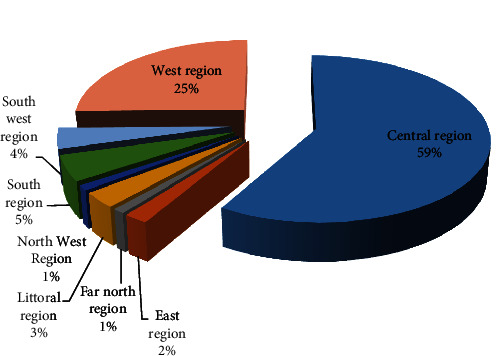
Distribution of research study on antiplasmodial activity in Cameroon.

Tables S2 and 1 show descriptive extracted data from the included studies in systematic review and meta-analysis, respectively. All studies were published between 2000 and 2021. More studies were conducted in the center region of Cameroon (*n* = 18) followed by the western region (*n* = 6). The rest of the plants were collected from South-west, Littoral, East, North-west, and Far north region. No plants were yet investigated in the North and Adamaoua regions (Figure 2). Almost 90 plants species (31 families) have so far been tested for their *in vitro* antiplasmodial activities against both/only resistant *P. falciparum* strains (W2, W2mef, INDO, W32, FCM29, FCB1, K1, NFS4, and Dd2) and/or susceptible strains (3D7, D-6, HB3, SHF4, and F32). Some studies (*n* = 16) had assessed the cytotoxicity of study plants. The cell model used for cytotoxicity was also different between the studies. Models used were U2OS, Hep G2, Hela, HFF, MRC-5, MRC-7, WI-38, HEK 239T, A375, WI-38, LLC-MK2, and RAW cell.

### 3.3. Qualitative Synthesis

#### 3.3.1. Acanthaceae

Stem bark of *Thomandersia hensii* was extracted with hexane, ethyl acetate, dichloromethane/methanol and methanol. They exerted an antiplasmodial activity against *Plasmodium falciparum* W2 strain with IC_50_ values of 53.9, 24.7, 77.2 and 68.2 *μ*g/ml respectively [31].

#### 3.3.2. Anacardiaceae

*Sorindeia juglandifolia* is a tree with 23 m height with no specific uses and no pharmacological studies so far [32]. However, Kamkumo et al. showed that a hexane/ethyl acetate extract of fruits of *S. juglandifolia* exerted inhibitory effects against *P. falciparum* W2 strain and recombinant falcipain-2, respectively. The IC_50_ values were 6.24 *μ*g/ml for W2 and 8.22 *μ*g/ml for falcipain-2 [32]. Secondary metabolites isolated, such as 2,3,6-trihydroxy benzoic acid and 2,3,6-trihydroxy methyl benzoate, demonstrated low inhibitory effects against *P. falciparum* strains, with IC_50_ values of 16.47 and 13.04 *μ*M against *P. falciparum* W2, and 35.41 and 6.09 *μ*M against falcipain-2, respectively [32]. Otherwise, aqueous and ethanol extract of bark or leaves of *Mangifera indica* exhibited a high selectivity index for their antiplasmodial activity (SI > 50) [21].

#### 3.3.3. Annonaceae

Several studies were performed to elucidate the pharmacological parameters of Annonaceae species commonly used in Cameroon against malaria and/or related symptoms. It was highlighted that methanol and/or ethanol extracts of *Annona muricata* (seeds), *Anonidium mannii* (leaves and twigs), *Polyalthia oliveri* (stem bark), *Polyalthia suaveolens* (twigs), *Uvariastrum zenkeri* (twigs), *Uvariopsis congolana* (stem), *Enantia chlorantha* (stem bark), *Xylopia aethiopica* (twigs, stem bark and roots), and *Xylopia Africana* (stem) exhibited antiplasmodial activities with IC_50_ values lower than 5 *μ*g/ml [33–35]. Moreover, both methanol and ethanol extracts of twigs of *Monodora myristica*, *Piptostigma calophyllum*, and *Uvariodendron molundense*, demonstrated an antiplasmodial activity with IC_50_ lower than 10 *μ*g/ml [33]. Similar results were found with the extracts of *Xylopia parviflora* (leaves and stem) and *Annona reticulate* (leaves) [33, 34]. *Uvaria banmanni* and *Uvariodendron calophyllum* did not have a high antiplasmodial activity (IC_50_ > 10 *μ*g/ml) [33]. A previous study, published by Boyom et al. showed that some acetogenin-rich extracts of *Uvariopsis congolana*, *Polyalthia oliveri*, and *Enantia chlorantha* exerted inhibitory effects against *P. falciparum* W2 strain [36]. The IC_50_ values of aqueous stem bark extracts of *Cleistopholis patens*, *Uvariastrum pierreanum, Xylopia phloiodora, Pachypodanthium confine, Xylopia aethiopica*, and *Hexalobus crispiflorus* were, respectively, 9.19, 6.08, 17.9, 16.6, 17.8, and 2.0 *μ*g/ml [36, 37]. Despite the good antiplasmodial activity of *H. crispiflorus*, few studies yet been done to isolate secondary metabolites responsible of this activity.

#### 3.3.4. Apocynaceae

Ndjakou Lenta et al. showed that the IC_50_ of methanol stem bark extract of *Rauvolfia macrophylla* was higher than 5 *μ*g/ml, and their selectivity index (SI) was greater than ninety [38]. Fotie et al. showed that stem bark extracts of *Holarrhena floribunda* exerted inhibitory effects against *P. falciparum* W2 and D-6 strains [39]. However, no evidence of antiplasmodial effect was shown with isolated secondaries metabolites [39]. The stem bark, the roots, the seeds, and the fruits of *Picralima nitida* are frequently used in Cameroonian traditional medicine to cure malaria or fever [31]. Only one study on the methanol extract of the stem bark of *P. nitida*, which showed the highest antimalarial activity (IC_50_ = 10 *µ*g/ml), has been performed [31]. The other extracts, (hexane and dichloromethane/methanol) showed low *in vitro* antimalarial activity against W2 strain [31]. Recently, Ma'mag et al. and Bitombo et al. highlighted the antiplasmodial activity of two Apocynaceae, *Funtunia elastica* [30], and *Tabernaemontana penduliflora* [40]. Methanol extract of *Funtunia elastica* exhibited a very high antiplasmodial activity against both Dd2 and 3D7 *P. falciparum* strains (IC50 < 5 *µ*g/ml). Terpenoid (3*β*-hydroxyurs-20(21)-en-29-oic acid, 2*α*,3*β*-hydroxyurs-20(21)-en-29-oic acid and 6′-O-acetylglucopyranosyl-3*β*-hydroxyurs-20(21)-en-29-oic acid) and alkaloid (1*α*, 11*α*, 17*α*-trihydroxy-3*β*-(N-benzamido)-5,6-dihydroantidysentericine) extracted from *Funtunia elastica* had a high selectivity index (SI > 37). The IC_50_ of hydroethanol extract of *Tabernaemontana penduliflora* was 15.76 and 18.46 *µ*g/ml, respectively, against Dd2 and 3D7 *P. falciparum* strains. Penduliflorine A/B and Tabernaemontine were the two best alkaloids extracted from *Tabernaemontana penduliflora* with IC_50_ < 5 *µ*g/ml.

#### 3.3.5. Asteraceae

The carrot-like tubers of *Vernonia guinensis* are commonly used in ethnomedicine. Toyang et al. investigated the antiplasmodial activity of crude extracts and pure compounds of *V. guinensis*. These pure compounds and crude extracts from *V. guinensis* inhibited the growth of HB3 and Dd2 [41]. The IC_50_ values of extracts were similar for HB3 and Dd2, and ranged from 1.64–27.2 *μ*g/ml for Hb3 and 1.82–30.0 *μ*g/ml for Dd2. The IC_50_ values of vernopicrin, vernomelitensin, and pentaisovalerylsucrose isolated from *V. guinensis* were similar to HB3 and Dd2 and ranged from 0.47–1.62 *μ*g/ml for HB3 and 0.57–1.49 *μ*g/ml for Dd2 [41]. Similar result was found with *Vernonia amygdalina* and *Vismia guinensis* which exhibited antiplasmodial activity without cytotoxicity [26].

#### 3.3.6. Bignoniaceae

The ethyl acetate extract of *Stereospermum zenkeri* has moderate activity against *P. falciparum* K1 chloroquine-resistant strain, with IC_50_ values below 10 *μ*g/ml [38]. However, it was been not clear if both ethyl acetate and methanol extracts of *Stereospermum acuminatissimum* could be active against *P. falciparum* K1 chloroquine-resistant strain (IC_50_ > 5 *μ*g/ml) [38]. Hexane and ethyl acetate extracts of *Markhamia tomentosa* [42] and *Kigelia africana* [43] exhibited a high antiplasmodial activity against W2 *P. falciparum* strain (IC50 < 5 *μ*g/ml).

#### 3.3.7. Burseraceae

Zofou et al. showed that leaves of *Dacryodes edulis* exibited an antiplasmodial activity against 3D7 strain of malaria parasite (IC_50_ = 6.45 *μ*g/ml) [26]. Also, sterm bark of *D. edulis* had an antiplasmodial activity against the same strain (IC_50_ = 4.34 *μ*g/ml) [28]. No sign of cytotoxicity was observed with extracts from *D. edulis* on LLC/MK2 epithelial cells [26, 28]. Apart from Afzelin, the selectivity index of all secondary metabolites from *D. edulis* (quercitrin, quercetin, methyl 3,4,5-trihydroxybenzoate and sitosterol 3-O-*β*-Dglucopyranoside sterol) were higher than 10.

#### 3.3.8. Caricaceae

Leaves of *Carica papaya* are constituents of Nefang, a traditional drug used to treat malaria in Cameroon. These leaves do not show any antiplasmodial activity against Dd2 strains of *P. falciparum* without toxicity [21].

#### 3.3.9. Celastraceae

Crude dichloromethane/methanol and secondary metabolites extracts from *Salacia longipes* exhibited a very high antiplasmodial activity against W2 *P. falciparum* strain [44]. And cytotoxicity analysis was performed.

#### 3.3.10. Clusiaceae

Ndjakou Lenta et al. tested fruits extracts and isolates compounds from *Pentadesma butyracea* for their antiplasmodial activity *in vitro* against the W2 strain chloroquine-resistant*P. falciparum* and other antimalarial drugs. Pericarp extract showed good antiplasmodial activity, with an IC_50_ of 1.83 *μ*g/mL, while the seed extract was inactive. Among all isolated compounds, only the xanthones exhibited antiplasmodial activity against the W2 strain, with garcinone *E* showing the best potency and followed by *α*-mangostin, cratoxylone, and pentadexanthone [45].

Previous study, performed by Zelefack et al., showed that isolated molecules from stem bark of *Pentadesma butyracea* cannot be lead candidates for treatment of malaria because of their high cytotoxicity. Therefore, they found that butyraxanthone A, butyraxanthone B, mangostanin, 1,3,6-trihydroxy-7-methoxy-2,8-diprenylxanthone, rubraxanthone, garcinone, gartanin, tovophyllin from the stem bark of *Pentadesma butyracea* showed good antiplasmodial activity [46].

Ndjakou Lenta et al. investigated three Clusiaceae, *Allanblackia monticola*, *Harungana madagascariensis*, and *Symphonia globulifera*. *Harungana madagascarensis* methanolic extract (IC_50_ = 3.6 *µ*g/mL) and *Symphonia globulifera* methanolic extract (IC_50_ = 4.1 *µ*g/mL) exhibited good antiplasmodial activity against *P. falciparum* K1 chloroquine-resistant strain, with IC_50_ values lower than 5 *µ*g/ml [38]. Whereas, they found that IC_50_ against *Plasmodium falciparum* K1 chloroquine-resistant strain of *Allanblackia monticola* was greater than 5 *µ*g/mL [38]. However, previous results of Azebaze et al. against *P. falciparum* FCM29 and F32 strain showed that IC_50_ of *Allanblackia monticola* methanolic extract was, respectively, 3.1 *µ*g/mL and 3.3 *µ*g/mL [47]. Three molecules extracted from *Allanblackia monticola* (Allanxanthone C, norcowanin, mangostin) exhibited good antiplasmodial activity [47].

#### 3.3.11. Combretaceae

Decoction extracts from the leaves of *Terminalia catappa* and leaves and bark of *Terminalia mantaly* exhibited very promising activity against *P. falciparum* 3D7 (IC_50_ = 2.49–6.41 *µ*g/mL) and *P. falciparum* INDO (IC_50_ = 1.90–8.10 *µ*g/mL) [22]. Moreover, Mbouna et al. showed that the aqueous extracts from leaf and stem bark of *Terminalia mantaly*, and the aqueous and methanolic extracts from leaf and root of *Terminalia superba* exhibited antiplasmodial activity [29].

#### 3.3.12. Ebenaceae

Methanol extract from powdered stem bark of *Diospyros sanza-minika* exhibited strong antiplasmodial effects with IC_50_ values of 1.7 against *P. falciparum* K1. The secondary metabolites isolated from the stem bark of *D. sanza-minika* were norbergenin, 4-O-galloylnorbergenin, 11-O-p-hydroxybenzoylnorbergenin, 4-O-(3′-methylgalloyl) norbergenin, and 4-Osyringoylnorbergenin. Norbergenin and 4-Osyringoylnorbergenin were found to be inactive, 4-O-galloylnorbergenin and 11-O-p-hydroxybenzoylnorbergenin showed moderate activity with IC_50_ values of 3.9 and 4.9 *μ*g/mL; 4-O-(3′-methylgalloyl)norbergenin showed the highest potency (IC_50_ value: 0.6 *μ*g/mL) [48].

#### 3.3.13. Euphorbiaceae

Ethyl acetate extracts of stems and twigs of *Alchornea lacifolia* displayed moderate antiplasmodial activity (IC_50_Pf3D7/INDO ranging 12.44–16.64 *µ*g/mL) against both *P. falciparum* strains, whereas the corresponding aqueous extracts were weakly active or inactive (>25 to >100 *µ*g/mL). Moreover, leaf and trunk extracts displayed weak antiplasmodial activity to inactivity against the sensitive and resistant *P. falciparum* strains [22]. Rufin Marie et al. also showed that the water maceration and decoction, and ethyl acetate extracts of leaves of *Drypetes principum* exhibited an antiplasmodial activity (IC_50_3D7/INDO = 4.91/6.64, 5.49/5.98, and 6.49/7.10 *µ*g/mL, respectively) [22]. Moreover, it was shown by Boyom et al. that the crude from the stem bark of *Croton zambesicus* and *Neoboutonia glabrescens* exhibited an antiplasmodial activity with an IC_50_ value of 5.69 g/ml and 5.50 g/ml, respectively [49]. However, aqueous extracts of *Antidesma laciniatum* did not show a good antiplasmodial activity (IC_50_ = 29.4 *µ*g/mL) [36]. Recently, Djouwoug et al. showed that Bridelia atroviridis exhibited a high antiplasmodial activity with SI = 12 [50].

#### 3.3.14. Fabaceae

Fabaceae extract was not widely investigated. The extracts of *Senna alata* were inactive [22].

#### 3.3.15. Guttiferaceae

Three species of Guttiferaceae (*Allanblackia floribunda, Allanblackia monticola*, and *Allanblackia gabonensis*) were tested for their antiplasmodial activity by Azebaze et al. [23, 47, 51]. They found that, *A. gabonensis* did not show any antiplasmodial activity. However, *A. floribunda* an *A. monticola* exhibited strong antiplasmodial effects. Macluraxanthone isolated from *A. floribunda* was the most active compound on two strains of Plasmodium followed by volkensiflavone with a IC_50_ of 0.46 and 0.99 *μ*g/mL for the F32 and 0.33 and 0.93 *μ*g/mL for the FcM29 strains respectively [51]. Allaxanthone B isolated from *A. monticola* was responsible of its antimalarial property with IC_50_ of 3.70 and 3.93 *μ*g/mL for the F32 and FcM29 strains respectively [51]. Five of other prenylated xanthones (*α*-mangosine, tovophiline A, allaxanthone C, rubraxanthone, norcowanine) isolated from *A. monticola* previously tested for antiplasmodial properties had displayed after 24 h of contact with the parasite a significant antiplasmodial activity (IC_50_ : 1.96–3.16 *µ*g/mL) on the F32 strain and (IC_50_ : 1.72–3.22 *µ*g/mL) on FcM29 [23, 47].

#### 3.3.16. Hypericaceae

Bazouanthrone and harunganin isolated from the root bark of *Harungana madagascariensis* were found to be active against W2 strain of *P. falciparum* with IC_50_ of 5.4 and 8.1 *µ*g/mL, respectively [52]. The hexane and ethanol extracts of the stem bark of *Psorospermum glaberrimum* showed good antiplasmodial activity against *P. falciparum* W2 strain, with IC_50_ of 0.87 and 0.95 *µ*g/mL, respectively [53]. Some isolated secondary metabolites (glaberianthrone, 3-geranyloxyemodin anthrone, friedelan-3-one, 3-prenyloxyemodin anthrone, acetylvismione D, betulinic acid, 2-geranylemodin, bianthrone 1a) from *Psorospermum glaberrimum* showed good antiplasmodial activity against *P. falciparum* W2 strain with 3acetylvismione D displaying the best potency (IC_50_ of 0.05 *µ*g/mL) [53]. Otherwise, two isolated secondary metabolites of *Hypericum lanceolatum* presented significant antiplasmodial activities (with IC_50_ < 5 *µ*g/mL) with 5-hydroxy-3-methoxyxanthone exerting the highest activity (IC_50_ of 3.26 *µ*g/mL), followed by betulinic acid (IC_50_ of 4.50 *µ*g/mL) [54].

#### 3.3.17. Lamiaceae

Neither *Ocimum basilicum* and *Ocimum canum,* previously found as a repellent, and nor *Ocimum gratissimum* which is part of Nefang (a traditional remedy usually used in Cameroon to treat malaria) showed antiplasmodial activity in vitro [21, 22, 55].

#### 3.3.18. Leguminoceae

Only *Kotschya speciosa* was investigated in this family and was not found to be active against *P. falciparum* [28].

#### 3.3.19. Loganiaceae

Tchinda et al. tested the stem bark of *Strychnos malacoclados.* They found that an ethyl acetate extract of this specie exhibited an antiplasmodial activity against the chloroquine-sensitive 3D7 strain of *P. falciparum* with IC_50_ of 2.85 *µ*g/ml [24]. All secondary metabolites extracted from *S. malacociados* displayed an antiplasmodial activity against the chloroquine-sensitive 3D7 strain of *P. falciparum* [24]. From the stem bark of *S. malacoclados*, one new bisindole alkaloid, 3-hydroxylongicaudatine Y, was isolated along with the known alkaloids vomicine, bisnordihydrotoxiferine, divarine, longicaudatine, longicaudatine Y, and longicaudatine F [24]. Strychnobaillonine from *Strychnos icaja* was found as a very high antiplasmodial compound with SI = 14 [56].

#### 3.3.20. Meliaceae

Happi et al. found that three secondary metabolites (prototiamins A, prototiamins B, prototiamins C, prototiamins *E*, prototiamins F, prototiamins G) extracted from *Entandrophragma congoënse* displayed significant *in vitro* antiplasmodial activity against the erythrocytic stages of chloroquinesensitive *P. falciparum* strain NF54 [57]. Prototiamin C was the most potent of the secondary metabolite isolated, with an IC_50_ value of 1.32 *µ*g/mL [57]. Several secondary metabolites (kotschyienone A, kotschyienone B, 7-deacetylgedunin, 7-deacetyl-7-oxogedunin, 3,6,8-trihydroxy-2-(3,4-dihydroxylphenyl)-4H-chrom-4-one, quercetin) isolated from *Pseudocedrela kostchyi* gave IC_50_ values ranging from 0.75 to 4.61 *μ*g/mL for antiplasmodial activity against chloroquine-sensitive (Pf3D7) and chloroquine-resistant (PfINDO) strains of *P. falciparum* [27]. However, extracts from *Entandrophragma angolense* and *Khaya grandifoliola* did not show antiplasmodial activity [31, 58, 59].

#### 3.3.21. Mimosaceae

The methanolic extract of Albizia zygia (IC_50_ = 1.0 *µ*g/ml) exhibited good antiplasmodial activity towards *P. falciparum* K1 chloroquine-resistant strain. However, cytotoxicity against L6 cell was found to be high (CC_50_ = 4.5 *µ*g/ml) [38].

#### 3.3.22. Monimiaceae

A phytochemical study of the methylene chloride/methanol extract of leaves of *Glossocalyx brevipes* afforded three metabolites (methyl 2-(1′*β*-geranyl-5′*β*-hydroxy-2′-oxocyclohex-3″-enyl) acetate, 2-(1′*β*-geranyl-5′*β*-hydroxy-2′-oxocyclohex-3″-enyl) acetic acid, liriodenine (alkaloid)) with modest in vitro activity against *P. falciparum* [60].

#### 3.3.23. Moraceae

Boyom et al.showed that, only methanol leaves extracts of *Artocarpus communis* among the three Moraceae studied samples (*Artocarpus communis*-stem bark and leaf, *Dorstenia convewa*-twigs) showed high potency against W2 *P. falciparumin vitro* with IC_50_ values below 5 g/ml [35]. Moreover, Ruffin Marie et al. and Mbosso et al. found that some species of Ficus (*Ficus benjamina*, *Ficus exasperate, Ficus elastic*) did not exhibit antiplasmodial activity [22, 61].

#### 3.3.24. Myrtaceae

*Psidium guajava* which is a constituent of Nefang exhibited an antiplasmodial activity with a very high selectivity index (SI > 77) [21]. Otherwise, *Eucalyptus globulus* did not exhibit good antiplasmodial activity [26].

#### 3.3.25. Olacaceae

Methanol extract of *Coula edulis* was found as effective against W2 and 3D7 *P. falciparum* strains (IC_50_ : 5.79 and 13.8 *μ*g/ml, respectively) with significant high selectivity index (SI > 10) [26].

#### 3.3.26. Pittosporaceae

It had been shown that stem bark extract (methanol and 1-O-[apha-L-(Rhamnopyranosyl]- 23-acetoxyimberbic acid 29-methyl ester) of *Pittosporum mannii* exhibited a very high antiplasmodial activity against K1 *P. falciparum* strain (IC50 : 4.3 and 1.02 *μ*g/ml respectively) [62].

#### 3.3.27. Poaceae

Arrey Tarkang et al. found that ethanol leaves extracts of *Cymbopogon citratus*, which is another constituent of Nefang, were not active against *P. falciparum* [21]. However, Akono Ntonga et al. showed that aqueous leaf extracts of this plant exhibited an antiplasmodial activity, with IC_50_ of 4.2 *µ*g/ml [55].

#### 3.3.28. Rubiaceae

*Schumanniophyton magnificum* and *Cuviera longiflora* did not show a good antiplasmodial activity *in vitro* [26, 31].

#### 3.3.29. Rutaceae

Ethanol leaves extracts of *Citrus sinensis* which equally constitute Nefang was not active against *P. falciparum* [21]. Moreover, Wansi et al. showed that, *Teclea afzelii* was not active against *P. falciparum* [63].

#### 3.3.30. Selaginellaceae

*Selaginella vogelli* was more toxic than active against *P. falciparum* [61].

#### 3.3.31. Zingiberaceae

Kenmogne et al. showed that some compound from seeds of *Aframomum zambesiacum* had a very low selectivity index (SI < 5) for their antiplasmodial activity [64].

### 3.4. Meta-Analysis

**Figure 3:**
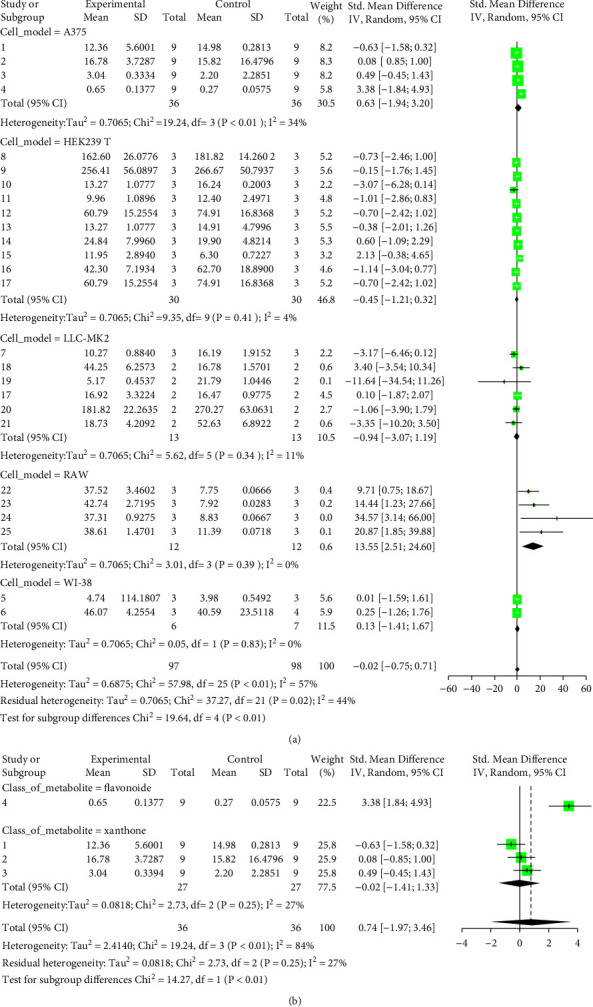
Results of the analysis of metabolites selectivity index to chloroquine resistant and susceptible strain, using random effect model.

**Figure 4:**
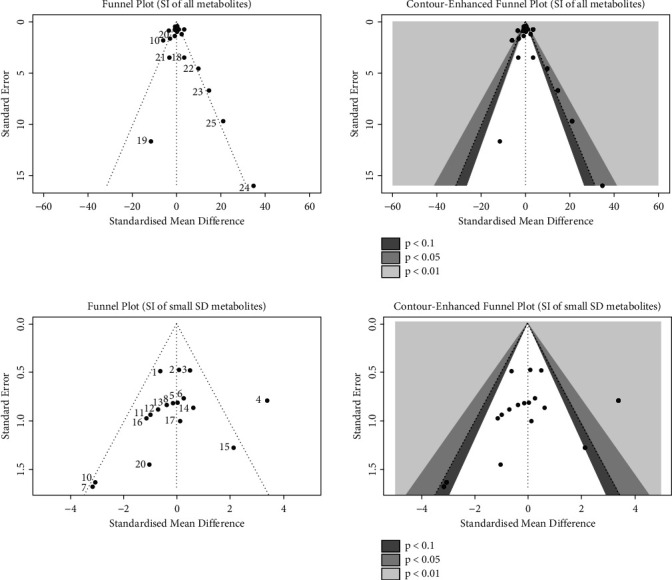
Funnel plots of meta-analysis of metabolites selectivity index.

We evaluated the impact of various potential interfering factors on the results of this meta-analysis. These included the part of the plant used, the nature of the crude extract, the strain of resistant or susceptible *P. falciparum* used, and the cell model used for cytotoxicity evaluation. Our results show that only the cell model used could lead to a significant heterogeneity (*p* < 0.01) between the different groups (Figure S1 and S3). The Asteraceae *V. amygdalina* and *V. guinensis* on the one hand and the Burseraceae *D. edulis* on the other hand, respectively, presented almost similar selectivity index in resistant and susceptible strains (*I*^2^: 0%; SMD: −1.60 and −0.06 respectively) (Figure 3). Meta-analysis of the antiplasmodial activity of various metabolites extracted from Cameroonian plants, highlights the strong antiplasmodial potential of metabolites **1, 2, 3, 5, 6, 7, 8, 9, 10, 11, 12, 13, 14, 16, 17, 19, 20,** and **21** (*I*^2^ < 30 and SMD <0.8) (Table 2; Figure 4). Eggers' test showed an asymmetry between the crude extract plant data (*P* = 0.289), suggesting a high risk of publication bias for these data (Table 3). In contrast, the secondary metabolite data did not show asymmetry (*P* = 0.048) suggesting a low risk of publication bias for these data (Table 3). Figure 4 shows the funnel plot of the selectivity index of the different secondary metabolites evaluated in these studies. The detailed analysis of this funnel plot shows a reservation on two of the compounds (**7** and **10**) previously considered as good candidates for their antiplasmodial activity (*P* < 0.1). Figure S2 shows the plants selectivity index to chloroquine resistant and susceptible strain, using random effect model.

## 4. Discussion

The fight against malaria is a great challenge characterized on the one hand by the resistance of the vector to the insecticides used and on the other hand by the resistance of the parasite to conventional drugs. It has to be noted that the discovery of new drugs against malaria is most often based on the results of research in natural pharmacopoeia as was the case for artemisinin and quinine [14, 15]. Some synthetic drugs such as dihydro-artemisinin and chloroquine are based on active ingredients from natural plants. Given the rise of resistance, especially to artemisinin and its derivatives used as the first line of defense against malaria, it is important to go back to the source of natural plants to look for potential candidates that could supplement this first line of defense while reducing the rapid emergence of resistance due to the massive use of a single type of drug. This review aimed at screening the antiplasmodial potential of Cameroonian plants and through a meta-analysis to bring out all the potential candidate active ingredients.

Taking into account the main goal of this systematic review, 42 *in vitro* studies were selected and 10 were submitted for meta-analysis. There is strong evidence that the dichloromethane extract of *Vernonia amygdalina* leaves [25], the dichloromethane and dichloromethane/methanol extracts of *Vismia guinensis* stem bark [26] and also the dichloromethane/methanol extracts of the leaves and stem bark of *Dacryodes edulis* [28] could be used as an antiplasmodial drug on chloroquine-sensitive and chloroquine-resistant strains (SI > 10). Despite the high risk of publication bias as revealed by Egger's test for crude extract results, the funnel plot showed us that studies on these above plants had a low risk of publication bias. Despite the fact that these plants had in common the type of extract used, our meta-analysis data did not support a significant effect of the type of extract used on the selectivity index of the plants for their antiplasmodial activities. The antiplasmodial potential of these plants would thus be particularly due to the nature of the active principles they contain. Indeed, the interactions between the compounds contained in the crude extracts of plants are often at the origin of a more or less high bioactivity of these crude extracts [65]. Antagonistic and synergistic interactions are the main causes. This review does not highlight the effect of these interactions on plant extracts, but we were able to demonstrate that 17 compounds extracted from plants, among which 5 (3 polyphenols and 2 terpenoids) extracts were from *Dacryodes edulis* [28], could be selected as ideal candidates for their antiplasmodial effect not only in chloroquine-sensitive strains, but even more so in chloroquine-resistant strains. *Dacryodes edulis* (Safou) is known for its dietary properties via its edible fruit; its curative and suppressive properties on a mouse model infected with *Plasmodium berghei* were demonstrated by maximum inhibition of Plasmodium at 57% and chemosuppression of the parasite at 71% [66]. In addition, this plant has antioxidant [67], anticancer [68], antidiabetic [69] properties that make it a good research model for its multiple effects. The limitation of this review is that it does not allow to conclude on the effect of these molecules on artemisinin-resistant strains which for several years has been considered as a first-line drug instead of chloroquine.

## 5. Conclusion

Despite the heterogeneity observed between the different plant families studied in Cameroon for their in vitro antiplasmodial effect, there is strong evidence that 17 active compounds from these plants would be ideal candidates for the synthesis of new antimalarial drugs. The *Dacryodes edulis* species, containing 5 of these active compounds, could be considered as a natural alternative in the treatment of uncomplicated malaria because of its inhibitory and suppressive capacities on the one hand and its relatively low cytotoxicity on the other hand.

## Figures and Tables

**Table 1:** Characteristic of studies included in meta-analysis.

**References**	**Plants**	**Family**	**Place of harvest**	**Part use**	**Extract**	**CQ R**	**CQ S**	**Cell model**
Tarkang et al. [21]	Mangifera indica	Anacardiaceae	Mballa II, Yaounde	Bark, leaves	Ethanol, Aqueous	Dd2	3D7	U2OS
	Psidium guajava	Myrtaceae	Nkomo, Yaounde	Leaves	Ethanol, Aqueous			
	Carica papaya	Caricaceae	Nkoabang, Yaounde	Leaves	Ethanol, Aqueous			
	Cymbopogon citratus	Poaceae	Kombone, Kumba	Leaves	Ethanol, Aqueous			
	Citrus sinensis	Rutaceae	Mamfe	Leaves	Ethanol, Aqueous			
	Ocimum gratissimum	Lamiaceae	Buea	Leaves	Ethanol, Aqueous			

Rufin et al. [22]	Alchornea Lacifolia	Euphorbiaceae	Mount Kalla	Twig, stem	Ethanol	INDO	3D7	HEK 239T
	Annona senegalensis	Annonaceae	Bafia	Bark, leaves	Ethanol, Hydroethanol			
	Annona senegalensis	Annonaceae	Bafia	Stem	Hydroethanol			
	Drypetes principum	Euphorbiaceae	Mount Kalla	Leaves	Decoction, Ethanol			
	Ficus benjamina	Moraceae	Yaounde	Leaves	Aqueous			
	Terminalia catappa	Combretaceae	Yaounde	Leaves	Decoction			
	Terminalia mantaly	Combretaceae	Yaounde	Leaves, bark	Decoction			

Azebaze et al. [23]	Allanblackia monticola	Guttiferaceae	Western region	Leaves	1, 2, 3, 4	FcM29	F32	A375

Tchinda et al. [24]	Strychnos malacoclados	Loganiaceae	Bertoua, Eastern region	Stem bark	5, 6	W32	3D7	WI-38

Zofou et al. [25]	Kigelia africana	Bignoniaceae	Bandjoun/West region	Stem bark	Hexane, Ethyl acetate, 7	W2	3D7	LLC-MK2
	Cuviera longiflora	Rubiaceae	Batcham/West region	Leaves	Dichloromethane/Methanol			

Zofou et al. [26]	Dacryodes edulis	Burseraceae	Batcham/West region	Leaves	Dichloromethane/Methanol	W2	3D7	LLC-MK2
	Eucalyptus globulus	Myrtaceae	Batcham/West region	Leaves	Dichloromethane/Methanol			
	Kotschya speciosa	Leguminoceae	Batcham/West region	Whole, aerial	Dichloromethane			
	Coula edulis	Olacaceae	Batcham/West region	Stem bark	Methanol			
	Vernonia amygdalina	Asteraceae	Batcham/West region	Leaves	Dichloromethane			
	Vismia guinensis	Asteraceae	Batcham/West region	Stem bark	Dichloromethane, Dichloromethane/Methanol			

Sidjui et al. [27]	Pseudocedrela kostchyi	Meliaceae	Karmai/Extreme Nord region	Roots	8, 9, 10, 11, 12, 13, 14, 15, 16, 17	INDO	3D7	HEK239T

Zofou et al. [28]	Dacryodes edulis	Burseraceae	Batcham/West region	stem bark	Dichloromethane/Methanol, 17, 18, 19, 20, 21	Dd2	3D7	LLC-MK2

Mbouna et al. [29]	Terminalia mantaly	Combretaceae	Yaoundé/Central region	Leaf, Stem bark, root	Aqueous, Methanol	INDO	3D7	HEK239T

Ma'mag et al. [30]	Funtumia elastica	Apocynaceae	Touessong, Center region	Leaves	Methanol, 22, 23, 24, 25	Dd2	3D7	RAW

**Table 2:** Molecules with the strong antiplasmodial potential activities isolated from Cameroonian pharmacopeia.

	**Metabolites**	**Structure**	**Class of metabolite**	**Family of metabolite**
**1**	***α*-Mangostin**	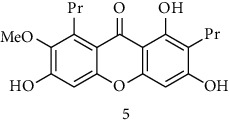	xanthone	Polyphenol

**2**	**Tovophyllin A**	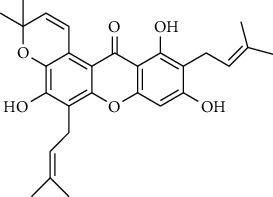	xanthone	Polyphenol

**3**	**1,7-Dihydroxy-3-methoxy-2-(3-methylbut-2-enyl) xanthone**	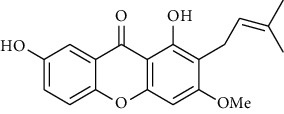	xanthone	Polyphenol

**4**	**Amentoflavone**	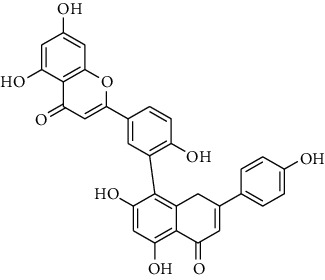	flavonoid	Polyphenol

**5**	**Longicaudatine**	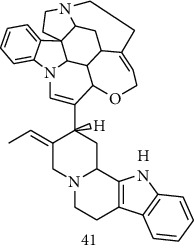	alkaloid	alkaloid

**6**	**Longicaudatine F**	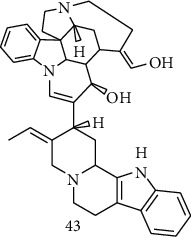	alkaloid	alkaloid

**7**	**Atranorin**	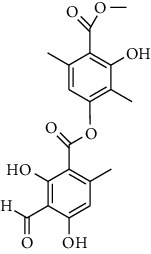	benzoic acid	Polyphenol

**8 & 9**	**kotschyienone A (R1 = R2 = H) and kotschyienone B (R1 = R2 = OH)**	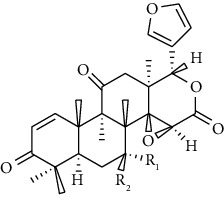	limonoid	terpenoid

**10**	**Andirobin**	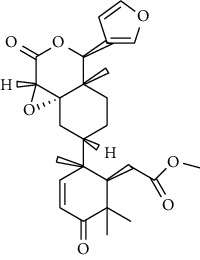	limonoid	terpenoid

**11**	**7-deacetylgedunin**	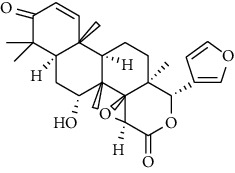	limonoid	terpenoid

**12**	**7-deacetyl-7-oxogedunin**	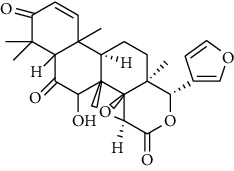	limonoid	terpenoid

**13**	***β*-sitosterol**	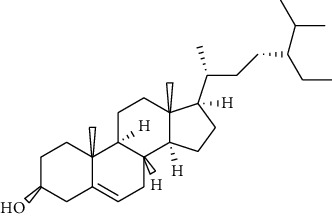	steroid	terpenoid

**14**	**stigmasterol**	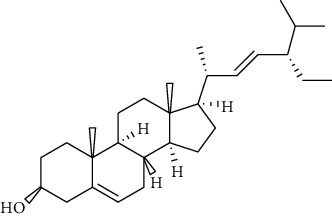	steroid	terpenoid

**15**	**betulinic acid**	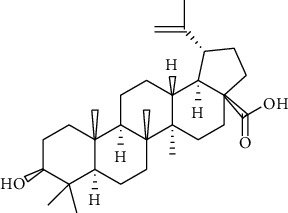	triterpene	terpenoid

**16**	**3,6,8-trihydroxy-2-(3,4-dihydroxylphenyl)-4H-chromen-4-one**	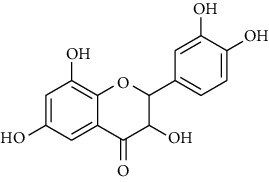	flavonoid	polyphenol

**17 & 18**	**Quercetin (R = H) And Quercitrin (R = Rha)**	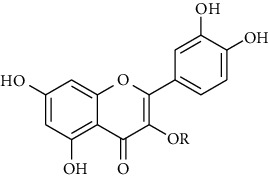	flavonoid	polyphenol

**19**	**Afzelin**	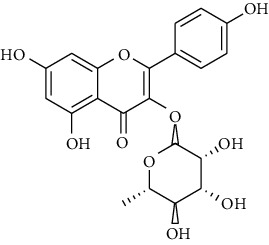	flavonoid	polyphenol

**20**	**methyl -3,4,5-trihydroxybenzoate**	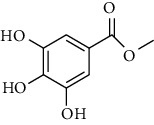	Benzoic acid	polyphenol

**21**	**sitosterol 3-O-*β*-Dglucopyranoside**	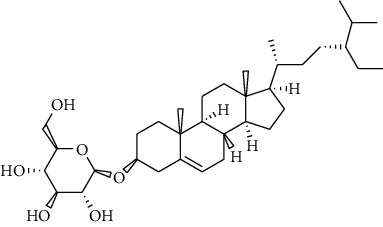	steroid	terpenoid

**Table 3:** Eggers' test of the intercept.

	Intercepyt	Confidence Interval	*t*	*p*
Metabolites	0.598	0.48–1.68	1.085	0.289
Plants	−0.758	−0.48–−1.69	−2.029	0.048

## Data Availability

All data generated or analyzed during this study are included within the article.

## Supplementary Materials

Supplementary Materials Figure S1. Analysis of potential confounding factors; Figure S2. Plants' selectivity index to chloroquine resistant and susceptible strain, using random effect model; Figure S3. Funnel plot for plants species; Table S1. Risk of bias assessment; Table S2. Characteristic of studies included in the systematic review.
