# Supplementary material for: The Antiplasmodial Potential of Medicinal Plants Used in the Cameroonian Pharmacopoeia: An Updated Systematic Review and Meta-Analysis
Source: Evid Based Complement Alternat Med. 2022 Oct 8;2022:4661753. doi: 10.1155/2022/4661753 (PMC9569203; doi:10.1155/2022/4661753)
Supplement: Supplementary Materials — Figure S1. Analysis of potential confounding factors; Figure S2. Plants' selectivity index to chloroquine resistant and susceptible strain, using random effect model; Figure S3. Funnel plot for plants species; Table S1. Risk of bias assessment; Table S2. Characteristic of studies included in the systematic review. [file 4661753.f1.zip › Table S1_Risk of bias assessment (1).docx]

| **Author** | **Year** | **C1** | **C2** | **C3** | **C4** | **C5** | **C6** | **C7** | **C8** | **Risk of bias** |
| --- | --- | --- | --- | --- | --- | --- | --- | --- | --- | --- |
| Akono et al | 2014 | Yes | Yes | Yes | No | No information | No information | No | NA | High |
| Tarkang et al | 2014 | Yes | Yes | Yes | Yes | Yes | No information | Yes | Yes | Low |
| Lenta et al | 2007 | Yes | Yes | No | No | Yes | No information | No | NA | High |
| Lenta et al | 2008 | Yes | Yes | Yes | No | No information | No information | No | NA | High |
| Rufin et al | 2018 | Yes | Yes | Yes | Yes | Yes | No information | Yes | Yes | Low |
| Bickii et al | 2006 | Yes | Yes | Yes | No | No information | No information | No | NA | High |
| Tangmouo et al | 2010 | Yes | Yes | Yes | No | Yes | No information | No | NA | Moderate |
| Azebaze et al | 2007 | Yes | Yes | No | No | Yes | Yes | No | NA | Moderate |
| Zofou et al | 2012 | Yes | Yes | Yes | Yes | No information | No information | No | Yes | Moderate |
| Kemgne et al | 2012 | Yes | Yes | Yes | No | No information | No information | No | NA | High |
| Toyang et al | 2013 | Yes | Yes | Yes | Yes | No information | No information | No | Yes | Moderate |
| Azebaze et al | 2015 | Yes | Yes | Yes | Yes | No information | No information | No | Yes | Moderate |
| Mbah et al | 2004 | Yes | Yes | No | Yes | No information | No information | No | Yes | High |
| Tchinda et al | 2012 | Yes | Yes | Yes | Yes | Yes | No information | Yes | Yes | Low |
| Tchinda et al | 2014 | Yes | Yes | No | No | Yes | No information | No | NA | High |
| Happi et al | 2015 | Yes | Yes | No | No | Yes | No information | No | NA | High |
| Lenta et al | 2011 | Yes | Yes | Yes | No | No information | No information | No | NA | High |
| Boyom et al | 2011 | Yes | Yes | Yes | No | No information | No information | No | NA | High |
| Tantangmo et al | 2010 | Yes | Yes | No | No | Yes | No information | No | NA | High |
| Boyom et al | 2003 | Yes | Yes | Yes | No | No information | No information | No | NA | High |
| Kamkumo et al | 2012 | Yes | Yes | No | Yes | No information | No information | No | Yes | High |
| Yamthe et al | 2015 | Yes | Yes | Yes | No | No information | No information | No | NA | High |
| Zofou et al | 2011 | Yes | Yes | Yes | No | Yes | No information | No | NA | Moderate |
| Bickii et al | 2000 | Yes | Yes | Yes | No | No information | No information | No | NA | High |
| Zofou et al | 2011 | Yes | Yes | Yes | Yes | Yes | No information | Yes | Yes | Low |
| Zofou et al | 2011 | Yes | Yes | Yes | Yes | Yes | No information | Yes | Yes | Low |
| Sidjui et al | 2018 | Yes | Yes | Yes | Yes | Yes | No information | Yes | Yes | Low |
| Fotie et al | 2006 | Yes | Yes | Yes | Yes | No information | No information | No | Yes | Moderate |
| Zofou et al | 2013 | Yes | Yes | Yes | Yes | Yes | No information | Yes | Yes | Low |
| Mbouna et al | 2018 | Yes | Yes | Yes | Yes | Yes | No information | Yes | Yes | Low |
| Boyom et al b | 2011 | Yes | Yes | Yes | No | No information | No information | No | No | High |
| Bickii et al b | 2006 | Yes | Yes | Yes | No | No information | No information | No | NA | High |
| Sjouwoug et al | 2021 | Yes | Yes | Yes | No | Yes | No information | No | NA | Moderate |
| Kenmogne | 2006 | Yes | Yes | Yes | No | Yes | No information | No | NA | Moderate |
| Nyongbela et al | 2013 | Yes | Yes | Yes | No | No information | No information | No | NA | High |
| Koagne et al | 2020 | Yes | Yes | No | No | No information | No information | No | NA | High |
| Ma'mag et al. | 2021 | Yes | Yes | Yes | Yes | Yes | No information | Yes | Yes | Low |
| Bitombo et al. | 2021 | Yes | Yes | Yes | Yes | No information | No information | No | Yes | Moderate |
| Mba'ning et al. | 2013 | Yes | Yes | Yes | No | No information | No information | No | NA | High |
| C1: Clear extraction method | | |  |  |  |  |  |  |  |  |
| C2: Appropriate in vitro method for antimalarial activity | | | | | | |  |  |  |  |
| C3: Appropriate number of replicate | | | | |  |  |  |  |  |  |
| C4: Resistant Vs Sensitive plasmodium strain comparison | | | | | | |  |  |  |  |
| C5: Appropriate in vitro method for cytotoxicity | | | | | | |  |  |  |  |
| C6: Culture of Plasmodium cells and control cells in the same condition | | | | | | | |  |  |  |
| C7: All required outcome (IC50, CC50 and SDs) available | | | | | | |  |  |  |  |
| C8: Quality control valid | | |  |  |  |  |  |  |  |  |
| NA: Not applicable | |  |  |  |  |  |  |  |  |  |
