# Supplementary material for: The Antiplasmodial Potential of Medicinal Plants Used in the Cameroonian Pharmacopoeia: An Updated Systematic Review and Meta-Analysis
Source: Evid Based Complement Alternat Med. 2022 Oct 8;2022:4661753. doi: 10.1155/2022/4661753 (PMC9569203; doi:10.1155/2022/4661753)
Supplement: Supplementary Materials — Figure S1. Analysis of potential confounding factors; Figure S2. Plants' selectivity index to chloroquine resistant and susceptible strain, using random effect model; Figure S3. Funnel plot for plants species; Table S1. Risk of bias assessment; Table S2. Characteristic of studies included in the systematic review. [file 4661753.f1.zip › Table S2_Characteristic of studies included in the systematic review (1).docx]

| **Author** | **Year** | **CQ Resistant** | **CQ Sensitive** | **Cell model** | **Place of harvest** | **Plant** | **Family** | **Part used** |
| --- | --- | --- | --- | --- | --- | --- | --- | --- |
| Akono et al | 2014 | FcB1 |  |  | Douala | Ocimum basilicum | Lamiaceae | Leaves |
|  |  |  |  |  |  | Ocimum canum | Lamiaceae |  |
|  |  |  |  |  |  | Cymbopogon citratus | Poaceae |  |
| Tarkang et al | 2014 | Dd2 | 3D7 | Hep G2, U2OS | Mballa II, Yaounde | Mangifera indica | Anacardiaceae | Bark, Leaves |
|  |  |  |  |  | Nkomo, Yaounde | Psidium guajava | Myrtaceae | Leaves |
|  |  |  |  |  | Nkoabang, Yaounde | Carica papaya | Caricaceae | Leaves |
|  |  |  |  |  | Kombone, Kumba | Cymbopogon citratus | Poaceae | Leaves |
|  |  |  |  |  | Mamfe | Citrus sinensis | Rutaceae | Leaves |
|  |  |  |  |  | Buea | Ocimum gratissimum | Lamiaceae | Leaves |
| Lenta et al | 2007 | K1 |  | L-6 | Bafoussam | Albizia zygia | Fabaceae | Stem bark |
|  |  |  |  |  | Bazou | Allanblackia monticola | Clusiaceae | Fruit |
|  |  |  |  |  | Bazou | Harungana madagascariensis | Hypericaceae | Seed |
|  |  |  |  |  | Bafoussam | Rauvolfia macrophylla | Apocynaceae | Stem bark |
|  |  |  |  |  | Bafoussam | Stereospermum acuminatissimum | Bignoniaceae | Stem bark |
|  |  |  |  |  | Bafoussam | Stereospermum zenkeri | Bignoniaceae | Stem bark |
|  |  |  |  |  | Bangangté | Symphonia globulifera | Clusiaceae | Stem bark |
| Lenta et al | 2007 | W2 |  |  | Bazou, Western region | Harungana madagascariensis | Hypericaceae | Root Bark |
| Lenta et al | 2008 | W2 |  |  | Ekombitié, Central region | Psorospermum glaberrimum | Hypericaceae | Stem bark |
| Rufin et al | 2018 | INDO | 3D7 | HEK 239T | Mount Kalla | Alchornea Lacifolia | Euphorbiaceae | Leaves, Twig, Stem, Trunk |
|  |  |  |  |  | Bafia | Annona senegalensis | Annonaceae | Bark, Leaves, Twig, Stem |
|  |  |  |  |  | Yaounde | Cananga odorata | Annonaceae | Flower |
|  |  |  |  |  | Mount Kalla | Drypetes principum | Euphorbiaceae | Leaves, Twig, Stem |
|  |  |  |  |  | Yaounde | Ficus benjamina | Moraceae | Fruit, Leaves, Stem |
|  |  |  |  |  | Yaounde | Ficus exasperata | Moraceae | Leaves, Stem |
|  |  |  |  |  | Yaounde | Occimum gratissimum | Lamiaceae | Leaves, Root, Stem |
|  |  |  |  |  | Yaounde | Senna alata | Fabaceae | Leaves, Stem, Twig |
|  |  |  |  |  | Yaounde | Terminalia catappa | Combretaceae | Leaves |
|  |  |  |  |  | Yaounde | Terminalia mantaly | Combretaceae | Leaves, Bark |
| Bickii et al | 2006 | W2 |  |  | Yokadouma, Eastern region | Entandrophragma angolense | Meliaceae | Stem bark |
|  |  |  |  |  | Eseka, Southern region | Picralima nitida | Apocynaceae | seeds |
|  |  |  |  |  | Ngomedzap, Southern region | Thomandersia hensii | Acanthaceae | Stem bark |
|  |  |  |  |  | Eseka, Southern region | Schumanniophyton magnificum | Rubiaceae | Stem bark |
| Tangmouo et al | 2010 | K1 |  | MRC-5 | Ngoumé, Central region | Diospyros sanza-minika | Ebenaceae | Stem Bark |
| Azebaze et al | 2007 | FcM29 | F32 | A375 | Western region | Allanblackia monticola | Guttiferaceae | Leaves |
| Zofou et al | 2012 | W2 | SHF4 |  | Bandjoun, Western region | Kigelia africana | Bignoniaceae | Stem bark |
| Kemgne et al | 2012 | W2 |  |  | Elounden, Central region | Uvariopsis congolana | Annonaceae | Stem, Leaves |
|  |  |  |  |  | Elounden, Central region | Polyalthia oliveri | Annonaceae | Stem bark |
|  |  |  |  |  | Elounden, Central region | Enantia chlorantha | Annonaceae | Stem bark |
|  |  |  |  |  | Elounden, Central region | Artocarpus communis | Moraceae | Stem bark, Leaves |
|  |  |  |  |  | Elounden, Central region | Dorstenia convexa | Moraceae | Twig |
|  |  |  |  |  | Elounden, Central region | Croton zambesicus | Euphorbiaceae | Stem bark |
|  |  |  |  |  | Elounden, Central region | Neoboutonia glabrescens | Euphorbiaceae | Leaves, Stem bark |
| Toyang et al | 2013 | Dd2 | Hb3 |  | Baicham, North West Region | Vernonia guineensis | Asteraceae | Root, Leaves |
| Azebaze et al | 2015 | FcM29 | F32 |  | Mt Kala, Central region | Allanblackia floribunda | Guttiferaceae | Leaves |
|  |  |  |  |  | Bangangté, Western Cameroon | Allanblackia monticola | Guttiferaceae | Leaves |
|  |  |  |  |  | Mt Kala, Central region | Allanblackia gabonensis | Guttiferaceae | Leaves |
| Mbah et al | 2004 | W2 |  |  | Kumba, South west region | Glossocalyx brevipes | Monimiaceae | Leaves |
| Tchinda et al | 2012 | W32 | 3D7 | WI-38 | Bertoua, Eastern region | Strychnos malacoclados | Loganiaceae | Stem bark |
| Tchinda et al | 2014 |  | 3D7 | WI-38 | Bertoua, Eastern region | Strychnos icaja | Loganiaceae | Root |
| Happi et al | 2015 | NF54 |  | L6 | Nkomokui, Central region | Entandrophragma congoënse | Meliaceae | Bark |
| Lenta et al | 2011 | W2 |  |  | Bazou, Western region | Pentadesma butyracea | Clusiaceae | Fruits |
| Boyom et al | 2011 | W2 |  |  | Mount Kalla, Central region | Cleistopholis patens | Annonaceae | Stem bark, Leaves |
|  |  |  |  |  | Mount Kalla, Central region | Uvariastrum pierreanum | Annonaceae | Stem bark, Leaves |
| Tantangmo et al | 2010 | W2, K1 |  | L6 | Mont Eloundem, Central region | Markhamia tomentosa | Bignoniaceae | stem bark |
| Mbosso et al | 2018 | 3D7 |  | HeLa | Yaoundé, Central region | Ficus elastica | Moraceae | aerial roots |
|  |  |  |  |  | Ngwei | Selaginella vogelli | Selaginellaceae | leaves |
| Boyom et al | 2003 | W2 |  |  | Mbalmayo, Central region | Xylopia phloiodora | Annonaceae | stem bark |
|  |  |  |  |  | Mbalmayo, Central region | Pachypodanthium confine | Annonaceae | stem bark |
|  |  |  |  |  | Mont Kalla, Central region | Antidesma laciniatum | Euphorbiaceae | leaves |
|  |  |  |  |  | Mbalmayo, Central region | Xylopia aethiopica | Annonaceae | stem bark |
|  |  |  |  |  | Mbalmayo, Central region | Hexalobus crispiflorus | Annonaceae | stem bark |
| Kamkumo et al | 2012 | W2 | 3D7 |  | Mont Kalla, Central region | Sorindeia juglandifolia | Anacardiaceae | Fruits |
| Zelefack et al | 2009 | FcB1-Columbia |  | MCF-7 | Bazou, West region | Pentadesma butyracea | Clusiaceae | stem bark |
| Yamthe et al | 2015 | W2 |  | HFF | Yaoundé, Central region | Annona muricata | Annonaceae | Pericarp, Stem bark |
|  |  |  |  |  | Yaoundé, Central region | Annona reticulata | Annonaceae | Leaf, Twig, Stem bark, Root, Fruit |
| Zofou et al | 2011 | W2mef |  | LLC-MK2 | Mount Bamboutos flanks/ West region | Hypericum lanceolatum | Hypericaceae | Stem bark |
| Bickii et al | 2000 | W2 |  |  | Foumban/West region | Khaya grandifoliola | Meliaceae | stem bark, Seeds |
| Zofou et al | 2011 | W2 | 3D7 | LLC-MK2 | Bandjoun/West region | Kigelia africana | Bignoniaceae | stem bark |
|  |  |  |  |  | Batcham/West region | Cuviera longiflora | Rubiaceae | Leaves |
|  |  |  |  |  | Batcham/West region | Dacryodes edulis | Burseraceae | Leaves |
|  |  |  |  |  | Batcham/West region | Eucalyptus globulus | Myrtaceae | Leaves |
|  |  |  |  |  | Batcham/West region | Kotschya speciosa | Leguminoceae | Whole plant, Aerial part |
|  |  |  |  |  | Batcham/West region | Coula edulis | Olacaceae | Stem bark |
|  |  |  |  |  | Batcham/West region | Vernonia amygdalina | Asteraceae | Leaves, Root bark |
|  |  |  |  |  | Batcham/West region | Vismia guinensis | Asteraceae | Stem bark |
| Sidjui et al | 2018 | INDO | 3D7 | HEK239T | Karmai/Extreme Nord region | Pseudocedrela kostchyi | Meliaceae | Roots |
| Fotie et al | 2006 | W2 | D-6 |  | Dja biosphere reserve/South region | Holarrhena floribunda | Apocynaceae | stem bark |
| Zofou et al | 2013 | Dd2 | 3D7 | LLC-MK2 | Batcham/West region | Dacryodes edulis | Burseraceae | stem bark |
| Mbouna et al | 2018 | INDO | 3D7 | HEK239T | Yaoundé/Central region | Terminalia mantaly | Combretaceae | Leaf, Stem bark, Root |
|  |  |  |  |  | Yaoundé/Central region | Terminalia superba | Combretaceae | Leaf, Stem bark, Root |
| Boyom et al | 2011 | W2 |  |  | Yaoundé/Central region | Annona muricata | Annonaceae | Leaves, Twigs, Flower parts, Pericarp, Pulp of fruit, Seeds |
|  |  |  |  |  | Yaoundé/Central region | Anonidium mannii | Annonaceae | Leaves, Twigs |
|  |  |  |  |  | Yaoundé/Central region | Monodora myristica | Annonaceae | Leaves, Twigs, Stem bark |
|  |  |  |  |  | Yaoundé/Central region | Piptostigma calophyllum | Annonaceae | Leaves, Twigs, Stem bark |
|  |  |  |  |  | Yaoundé/Central region | Polyalthia oliveri | Annonaceae | Leaves, Twigs, Stem bark |
|  |  |  |  |  | Yaoundé/Central region | Polyalthia suaveolens | Annonaceae | Leaves, Twigs, Stem bark |
|  |  |  |  |  | Yaoundé/Central region | Uvaria banmanni | Annonaceae | Leaves, Twigs |
|  |  |  |  |  | Yaoundé/Central region | Uvariastrum zenkeri | Annonaceae | Leaves, Twigs |
|  |  |  |  |  | Yaoundé/Central region | Uvariodendron calophyllum | Annonaceae | Leaves, Twigs |
|  |  |  |  |  | Yaoundé/Central region | Uvariodendron molundense | Annonaceae | Leaves, Twigs |
|  |  |  |  |  | Mt Kalla/Central region | Xylopia aethiopica | Annonaceae | Leaves, Twigs, Stem bark, roots |
|  |  |  |  |  | Mt Kalla/Central region | Xylopia africana | Annonaceae | Leaves, Stem |
|  |  |  |  |  | Mt Kalla/Central region | Xylopia parviflora | Annonaceae | Leaves, Stem, Seeds |
| Bickii et al | 2006 | W2 |  |  | Awae forest/Central region | Entandrophragma angolense | Meliaceae | Stem bark |
| Sjouwoug et al | 2021 | Dd2 |  |  | Mbalmayo, Central region | Bridelia atroviridis | Euphorbiaceae | Stem bark |
| Kenmogne | 2006 | Dd2 |  |  | Nyassosso, South-west | Aframomum zambesiacum | Zingiberaceae | Seeds |
| Nyongbela et al | 2013 | K1 |  |  | Bali Nyonga, Littoral | Pittosporum mannii | Pittosporaceae | Stem bark |
| Koagne et al | 2020 |  | 3D7 | HepG2 | Santchou, West region | Albizia zygia | Mimosaceae | Leaves |
| Ma'mag et al. | 2021 | Dd2 | 3D7 | RAW | Touessong, Center region | Funtumia elastica | Apocynaceae | Leaves |
| Bitombo et al. | 2021 | Dd2 | 3D7 |  | Mbalmayo, Central region | Tabernaemontana penduliflora | Apocynaceae | Bark |
| Mba'ning et al. | 2013 | W2 |  |  | Mount Kala, Centre region | Salacia longipes | Celastraceae | Seeds, Pericarp |
