# Supplementary figures and images for: The Antiplasmodial Potential of Medicinal Plants Used in the Cameroonian Pharmacopoeia: An Updated Systematic Review and Meta-Analysis
Source: Evid Based Complement Alternat Med. 2022 Oct 8;2022:4661753. doi: 10.1155/2022/4661753 (PMC9569203; doi:10.1155/2022/4661753)

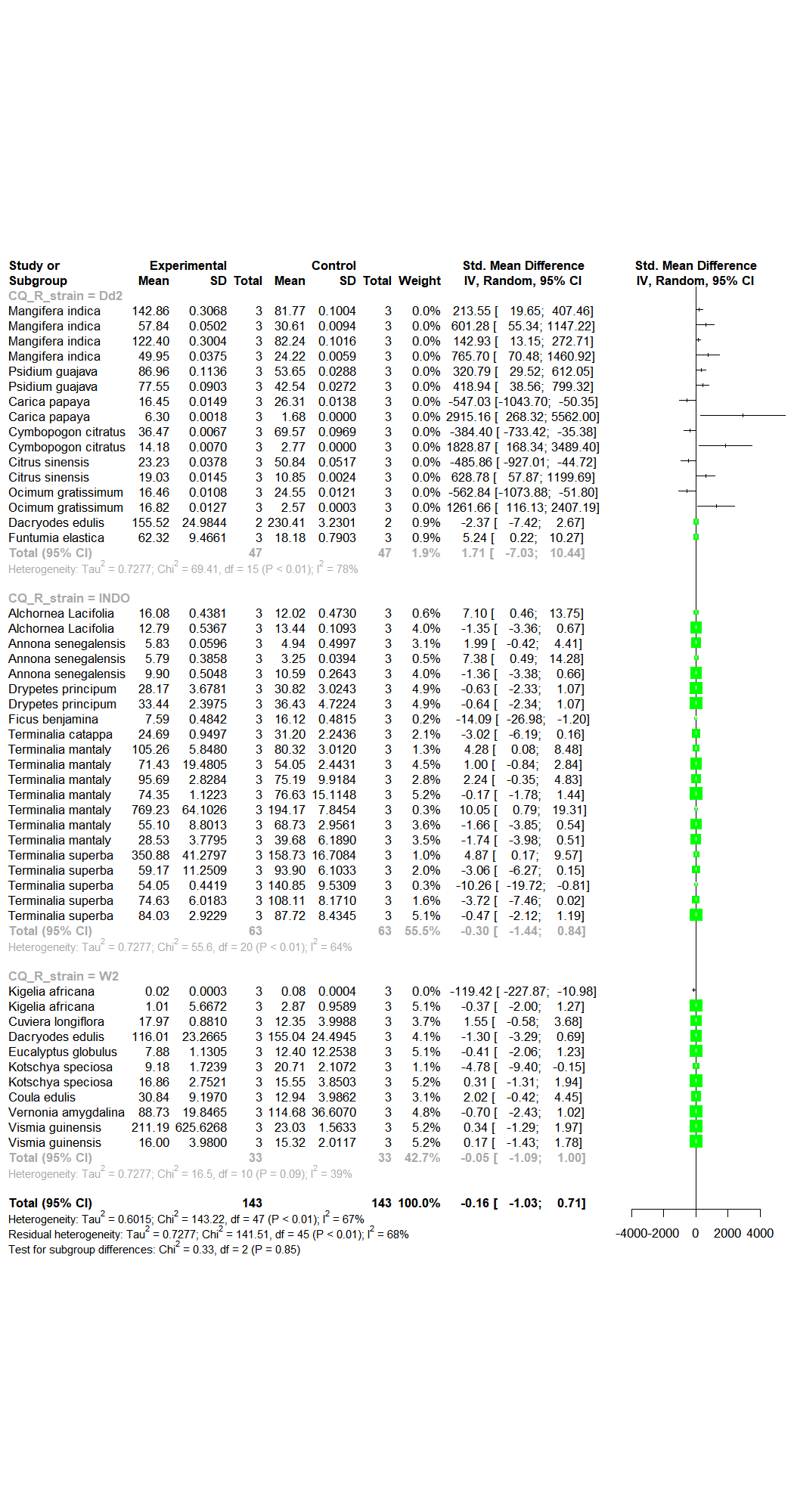


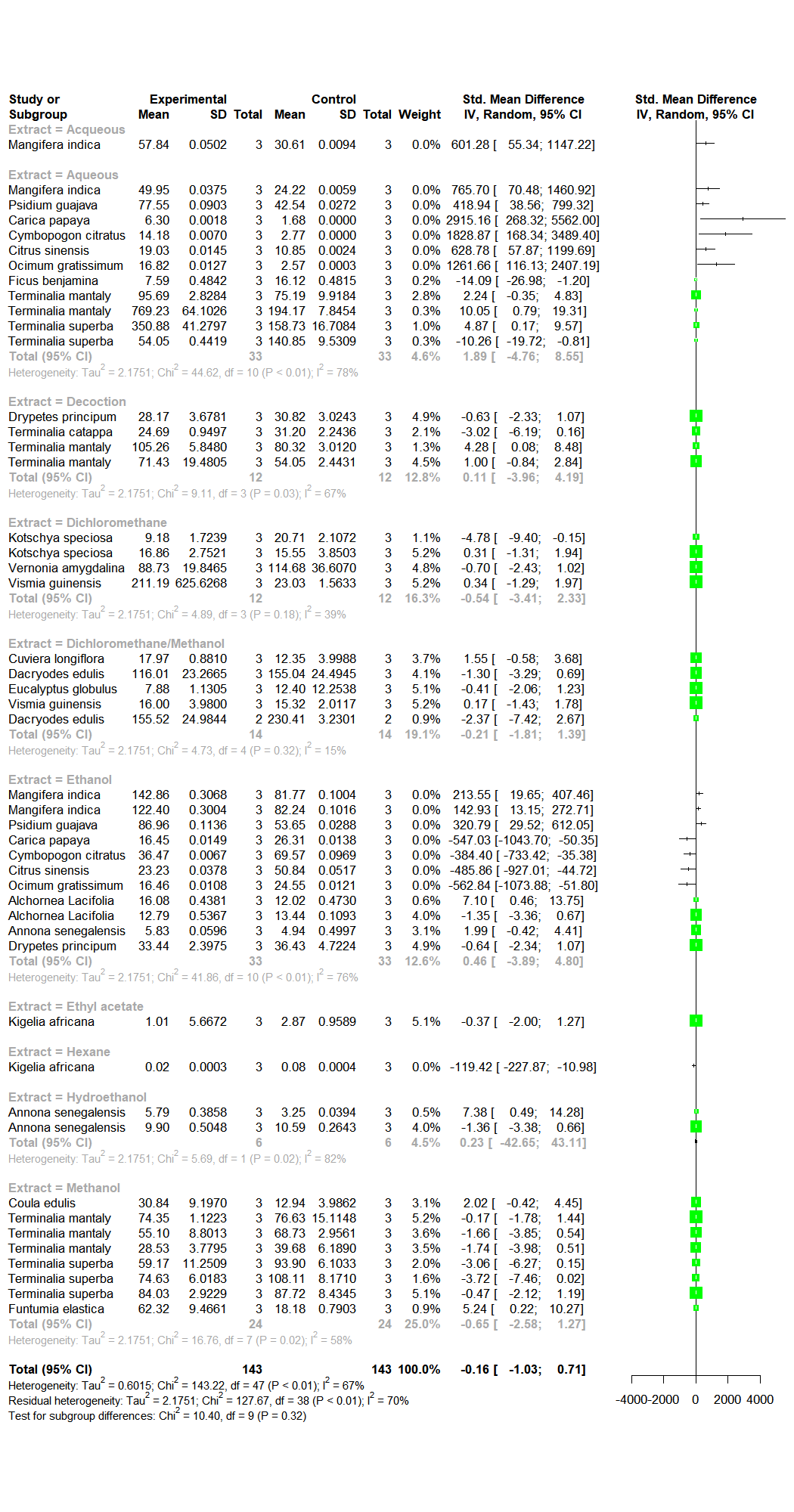


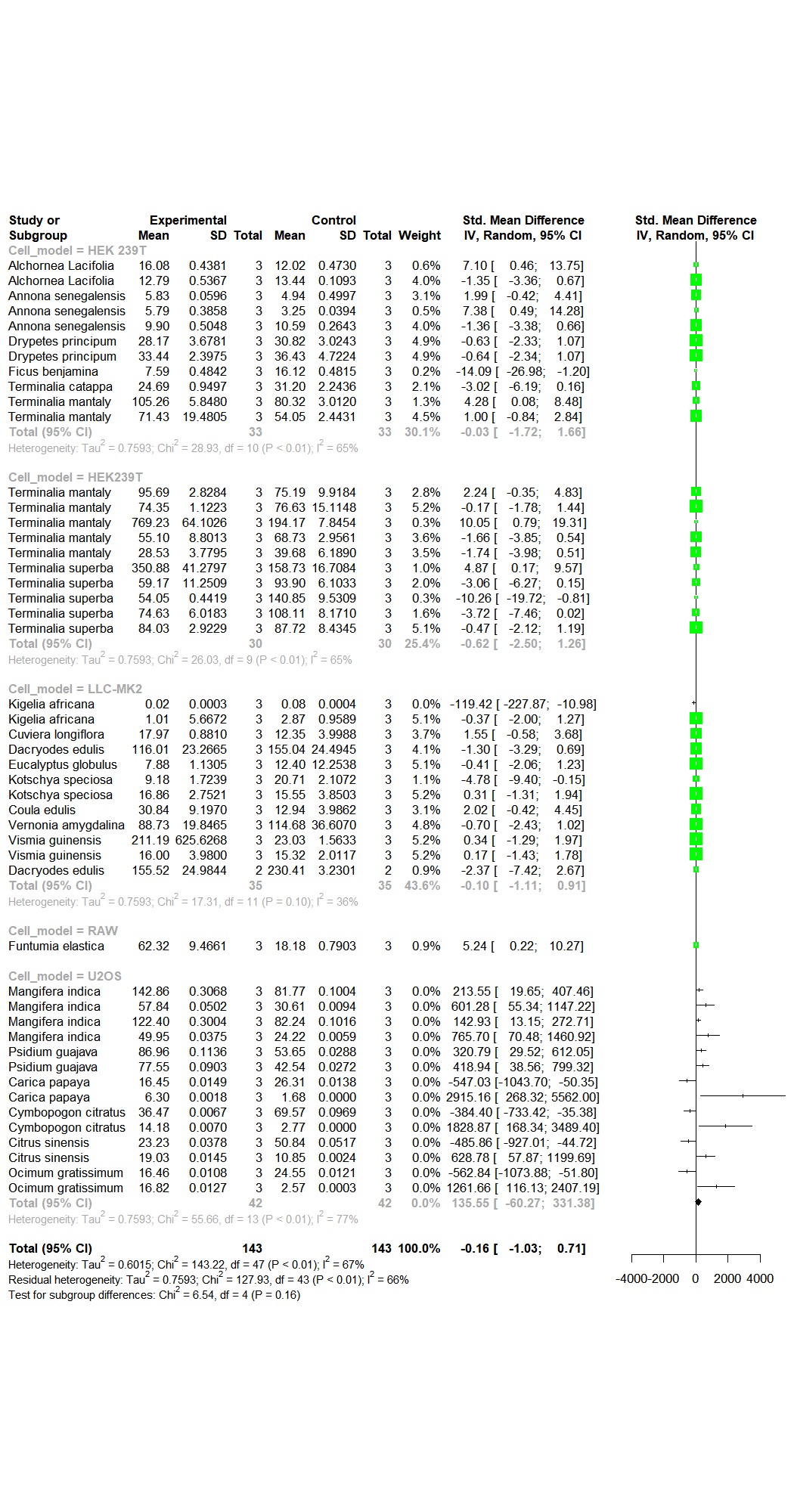


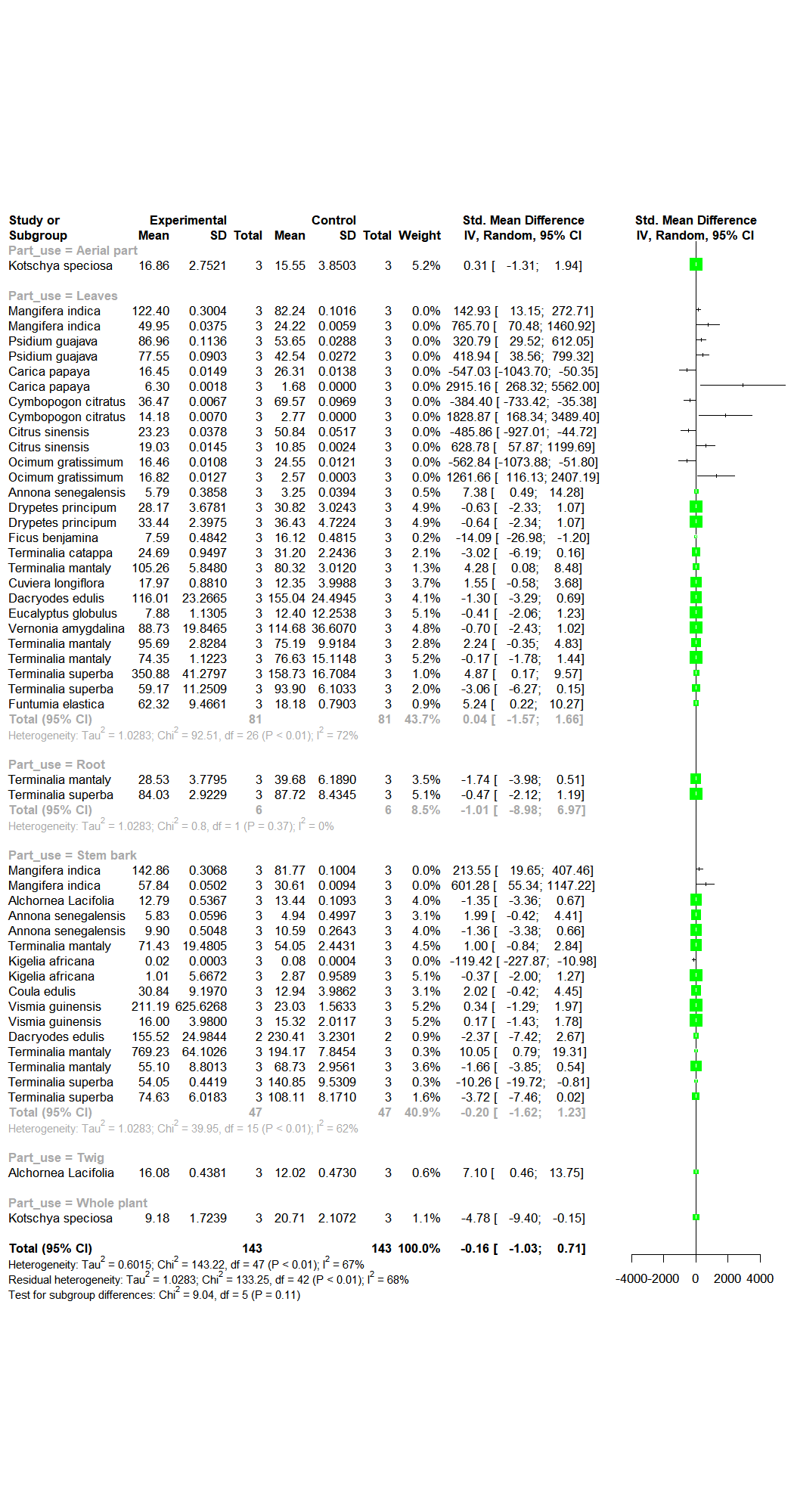


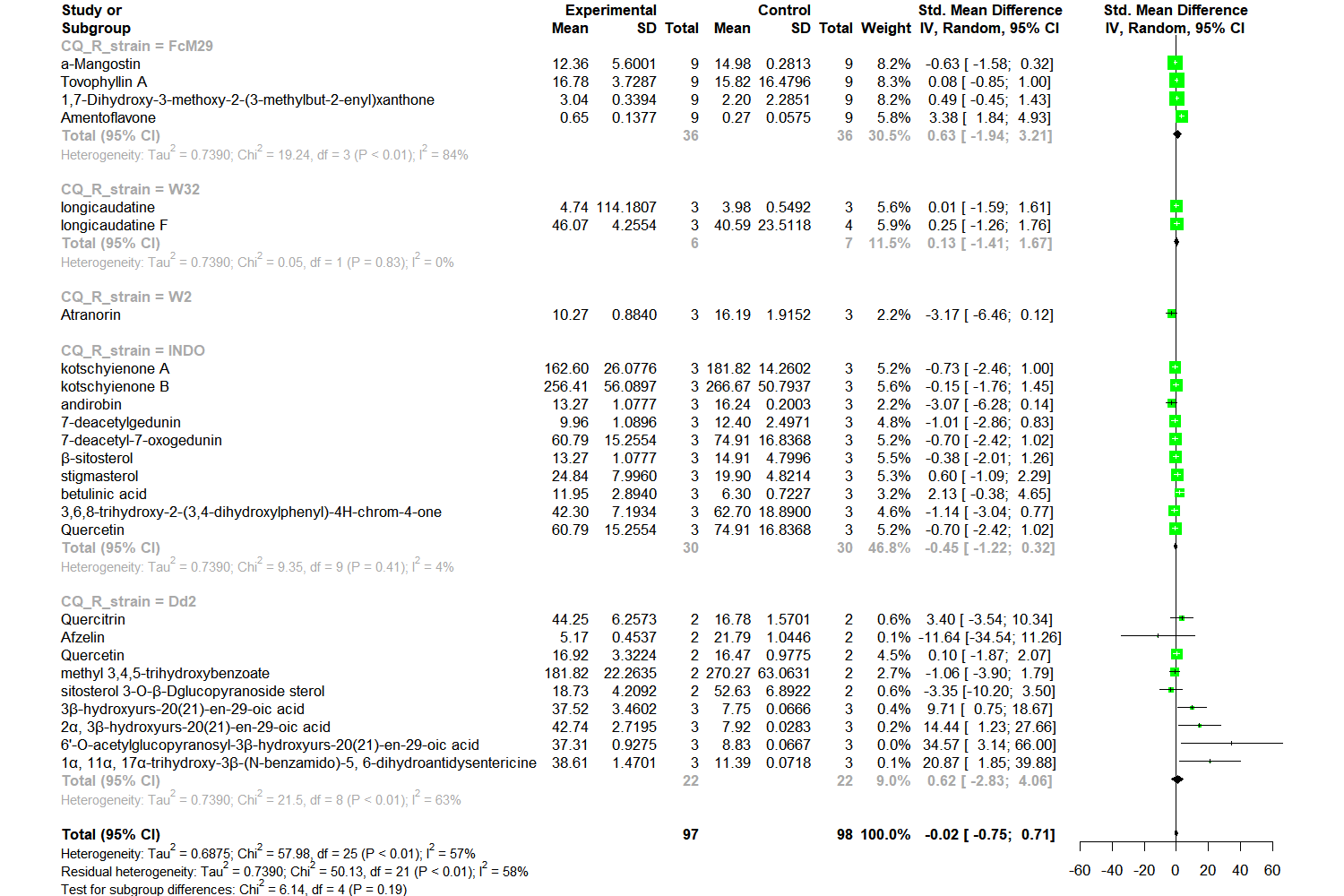


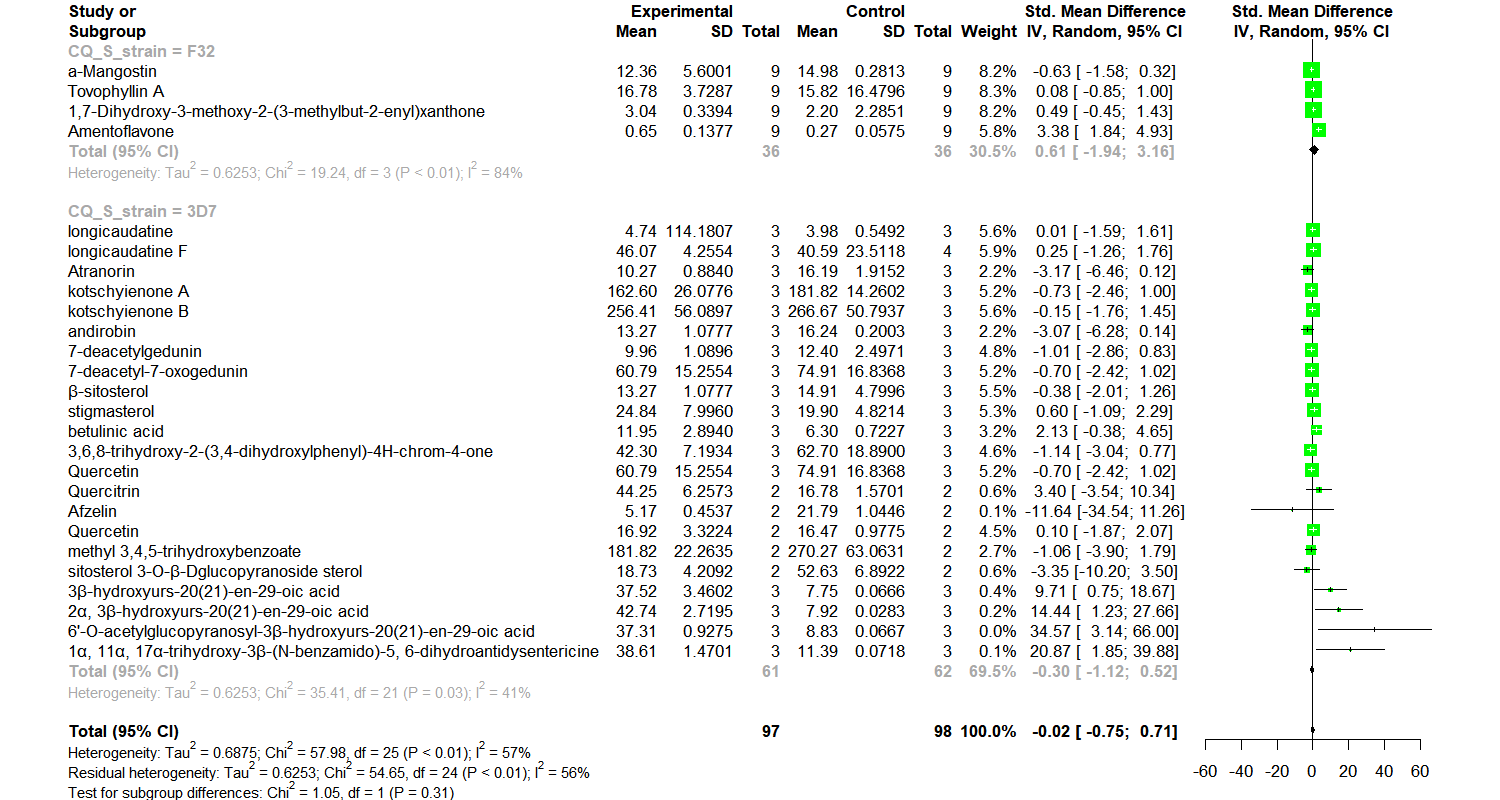

Supplement: Supplementary Materials — Figure S1. Analysis of potential confounding factors; Figure S2. Plants' selectivity index to chloroquine resistant and susceptible strain, using random effect model; Figure S3. Funnel plot for plants species; Table S1. Risk of bias assessment; Table S2. Characteristic of studies included in the systematic review. [file 4661753.f1.zip › Figure S1_ Analysis of potential confounding factors (1).docx]

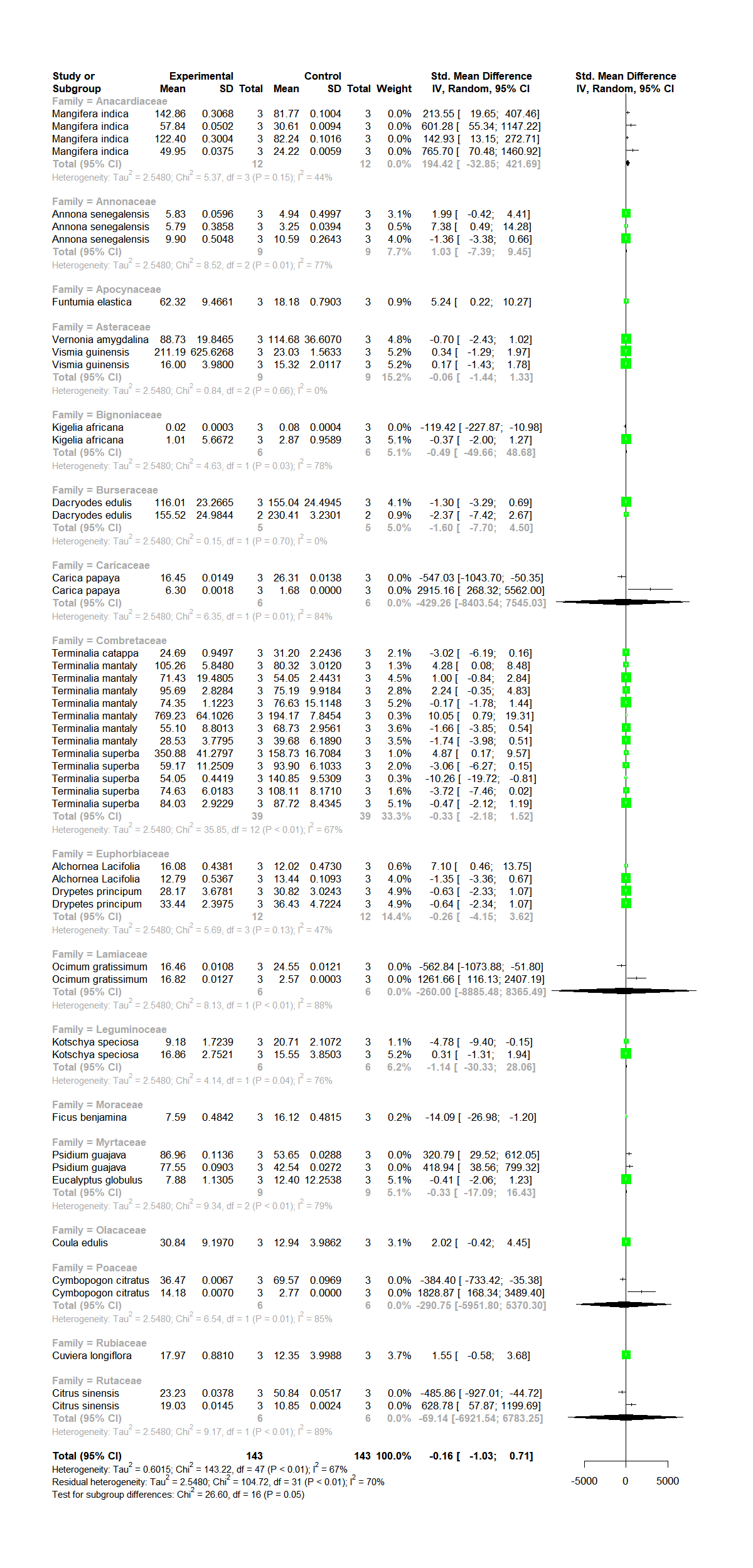

Supplement: Supplementary Materials — Figure S1. Analysis of potential confounding factors; Figure S2. Plants' selectivity index to chloroquine resistant and susceptible strain, using random effect model; Figure S3. Funnel plot for plants species; Table S1. Risk of bias assessment; Table S2. Characteristic of studies included in the systematic review. [file 4661753.f1.zip › Figure S2_ plants selectivity index to chloroquine resistant and susceptible strain, using random effect model (1).docx]

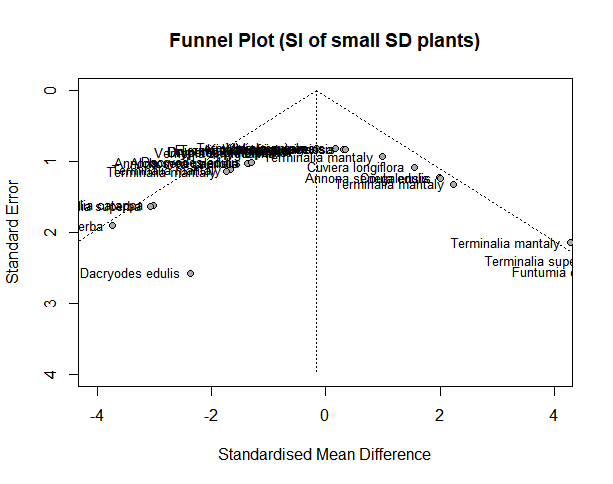


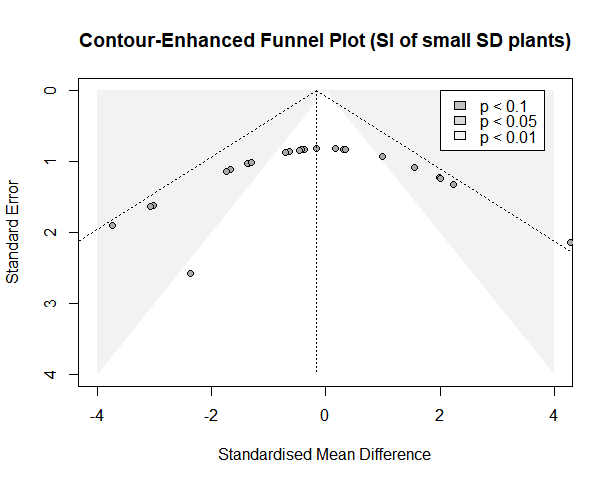

Supplement: Supplementary Materials — Figure S1. Analysis of potential confounding factors; Figure S2. Plants' selectivity index to chloroquine resistant and susceptible strain, using random effect model; Figure S3. Funnel plot for plants species; Table S1. Risk of bias assessment; Table S2. Characteristic of studies included in the systematic review. [file 4661753.f1.zip › Figure S3_ Funnel plot for Plants species (1).docx]
